# Clinical characteristics and quality of life in women with COPD: an observational study

**DOI:** 10.1186/1472-6874-14-31

**Published:** 2014-02-20

**Authors:** Chantal Raherison, Isabelle Tillie-Leblond, Anne Prudhomme, Camille Taillé, Elisabeth Biron, Cecilia Nocent-Ejnaini, Benigne Mathieu, Juliette Ostinelli

**Affiliations:** 1 Department of Respiratory Diseases, CHU Bordeaux, U897, ISPED, University of Bordeaux Segalen, Bordeaux, France; 2 Division of Pulmonary Medicine and Immuno-Allergy, CHRU Lille, Lille, France; 3 Division of Pulmonary Medicine, Bigorre Hospital, Tarbes, France; 4 Université Paris Diderot, Service de Pneumologie et Centre de Compétence des Maladies Pulmonaires Rares, Paris, France; 5 Assistance Publique-Hôpitaux de Paris, Hôpital Bichat, Paris; INSERM U700, Paris, France; 6 Jean Mermoz Hospital, Lyon, France; 7 Hospital of the Basque Coast, Bayonne, France; 8 Astra Zeneca, Rueil Malmaison, Paris, France

**Keywords:** COPD, Gender, Quality of life, Comorbidities, Chronic sputum

## Abstract

**Background:**

The impact of COPD on patient’s quality of life is well established, but gender differences have received little attention.

**Methods:**

To describe factors associated with the health-related quality of life by gender: A cross-sectional observational study (NCT01007734) was conducted in COPD patients followed by pulmonologists. The first patient included had to be a woman. Data concerning the patient, COPD and their management were collected by the physician. The patient had to fill in several questionnaires: Saint-George Hospital respiratory Questionnaire (SGRQ-C), and motivation to quit smoking.

**Results:**

Four hundred and thirty patients were included: mean age 63.9 ± 11.3 years; 57.4% were women. Women were significantly younger than men (61.9 vs. 66.6) and their tobacco use was lower (37.1 vs. 40.4 PY). Cardiovascular comorbidities were more frequent in men while osteoporosis, anxiety and depression were frequent in women. The frequency of cough, sputum and the severity of dyspnea did not differ significantly between genders. Lung function impairment was less severe in women than in men (mean FEV1 52% predicted normal vs. 47. 8%). Anxiety score was higher (score 9.8 vs. 7.1) and quality of life (SGRQ-C) more impaired in women (scores 50.6 vs. 45.4; p < 0.02) than in men. Moreover, in multivariate analysis, chronic sputum was associated with higher SGRQ-C scores in women but not in men.

**Conclusions:**

This study underlines that despite less airflow limitation, quality of life is more impacted by chronic sputum in women than in men.

## Background

According to WHO, chronic obstructive pulmonary disease (COPD) is now the fourth leading cause of death and will become the third in 2020. Some studies have shown that prevalence of COPD is increasing faster among women than men with different geographic distributions. In industrialized countries, COPD is usually associated with the increase in active smoking in women [1,2], probably with an underestimation of the impact of working exposure [3]. In the developing countries, the impact of COPD seems to be more associated with exposure to biomass in women [4].

Although COPD is the consequence of an interaction between genetic predisposition and environmental exposure, the influence of gender on the clinical expression of COPD has received limited attention. In general practice, COPD in women who smoke is often underdiagnosed [5,6]. Previous studies suggested that for the same severity of the disease, women with COPD seem to report more dyspnea and worse quality of life than men.

Quality of life is now a major component of the management of the disease, and valid criteria are needed to assess the efficacy of medication. Health status measurement is becoming an important issue for the day-to-day management of COPD patients in both primary and secondary health care [7]. Studies report that a shorter survival is related to worse health status/HRQoL (Health-related Quality of Life). Since health status is considered a major goal in managing the disease, physicians should focus on improving it. In addition, previous studies suggested that determinants of quality of life related to respiratory status differ by gender [8].

The first objective of our study (NCT01007734) was to describe the main factors determining the quality of life related to respiratory status according to gender in patients with moderate-to-severe COPD. The second objective was to determine the characteristics of COPD by gender after adjusting on age and active smoking.

## Methods

This was a multicenter, descriptive, cross-sectional study conducted in France from September 2009 to November 2010.

Physicians were recruited by telephone at random among a database of French pulmonologists. Each pulmonologist recruited at least three consecutive patients once the first woman was included. Inclusion criteria were outpatients more than 40 years old, with a spirometric diagnosis of COPD according to GOLD guidelines with post-bronchodilator FEV1/FVC under 0.70 and FEV1 under 80% predicted. Patients might have been followed for COPD for at least three months. They provided written informed consent and had to be able to complete the Saint **G**eorge **H**ospital **R**espiratory **Q**uestionnaire (SGRQ).

Exclusion criteria were as follows: recent exacerbation within 6 weeks, current or history of asthma, patients with long-term requirement of oxygen, non-invasive ventilation or tracheotomy, or participating in another trial. The physician filled in a medical questionnaire with demographics, comorbidities, COPD description, and COPD treatment concerning the patient. Chronic cough was defined as cough during 3 months and during two consecutive years, which the admitted definition of chronic bronchitis, the same definition was made for chronic sputum.

The BOD index was made with Body mass (<21 kg/m2), Obstruction (FEV1) and level of Dyspnea (MRC) without 6 minutes walking test (not available). Patients completed the following self-administered questionnaires, the (**SGRQ-C**), the **H**ospital **A**nxiety and **D**epression **S**cale (**HADS**) and the **M**otivation to quit smoking (**Q-MAT**, active smokers only).

The SGRQ is scaled from 0 (optimal health) to 100 (worst health) [9]. A total score and three sub-scores (symptoms, activities, impacts) are calculated.

The French translated version of SGRQ had been studied by Bourbeau et al. [10] and showed higher test-retest reliability (0.80).

The HADS is scaled [11] from 0 (no anxiety, no depression) to 21 (severe anxiety, or depression) for each subscale, French version used since 1985 [12].

The Q-MAT [13] is scaled from 0 (lack of motivation) to 20 (excellent motivation).

### Statistical analysis

Descriptive statistics (qualitative and quantitative data) were performed at baseline. We compared for clinical components and quality of life, men and women, then and after including significant factors, a multivariate model was used to determine factors related to quality of life, specifically for men and women, by stratifying by gender. Correlations were determined using Spearman correlation. Then, we correlated SGRQ total score with different variables using a multiple linear regression analysis using backward Wald criteria (p < 0.20), with those variables that significantly correlated with total SGRQ score. The criterion for statistical significance was 0.05. The software program used was SAS version 8.2.

The protocol was approved in France by the Comité Consultatif sur le Traitement de l’Information en matière de Recherche dans le domaine de la Santé (CCTIRS) and the Commission National Computer and Freedoms (CNIL). All patients were informed about the objectives of the study, and agreed to participate and fill in the questionnaires. The study was conducted according to the recommendations of the declaration of Helsinki for investigation with human subjects.

## Results

One hundred and forty-six physicians included 446 patients, among them 430 had data available for analysis. Mean age of physicians was 51.9 years (SD 7.9) and 77.3% of them were men. 60% of them were private practitioners and 40% were employed by hospitals.

Among the patients, 57.4% of them were female and 42.6% male. Descriptive analysis (Table 1) showed that women with COPD were younger, had lower BMI, and were more un-employed and lived alone more than men. Men were more frequently smokers or ex smokers than women (97.8% versus 84. 6% p < 0.001) but frequency of active smoking was higher in women, with a significant difference in the amount of smoking. Cardiovascular comorbidities, dyslipidemia, alcoholism and sleep apnea syndrome were more frequent in men. By contrast, osteoporosis, past anxiety and depression were more frequent in women.

**Table 1:** Descriptive characteristics and co-morbities by gender

	**Men (n = 183) 42.6%**	**Women (n = 247) 57.4%**	**p**	**n (M/W)**
**Mean age (years)**	66.6 ± 10.8	61.9 ± 11.3	<0.001	175/242
**% of subjects BMI <21 (Kg/m**^**2**^**)**	14.9	29.5	0.002	102/147
**Living alone at home (%)**	17.6	36.1	<0.001	181/244
**Unemployed**	11.8	25.2	<0.001	170/238
**Non-smoker/current smokers (%)**	2.2/30.2	15.4/44.1	<0.001	182/247
**Tobacco use (pack- years)**	40.5 ±19	37 ±16	0.041	170/203
**Comorbidities (%)**				
**Arterial hypertension**	41.4	32.9	NS	181/246
**Ischemic heart disease**	24.4	5.7	<0.001	180/245
**Arteriopathy**	12.2	3.7	0.001	181/245
**Heart failure**	8.4	2.9	0.011	178/245
**Diabetes**	11.5	6.5	NS	182/246
**Dyslipidemia**	26	16.7	< 0.02	181/246
**Alcoholism**	8.8	3.3	0.013	181/246
**Sleep apnea syndrome**	13.3	4.1	0.001	180/245
**Osteoporosis**	2.8	19.6	<0.001	178/240
**Anxiety disorder**	16.8	29.4	0.003	179/245
**Depression disorder**	4.6	19	<0.001	175/242

There was no difference between men and women regarding the frequency of cough and sputum and the severity of dyspnea (Table 2). Women seemed to have more exacerbations than men but the difference was not statistically significant. Regarding the severity of COPD according to the GOLD classification, there was a higher proportion of stage 2 and less stage 4 in women compared to men. Women used more short-acting ß2-agonists, with no difference for other inhaled treatment for COPD. There was a higher proportion of women with anxiety as measured by the HAD questionnaire compared to men.

**Table 2:** Clinical description of COPD by gender

	**Men (n = 183)**	**Women (n = 247)**	**p**	**n (M/W)**
**COPD symptoms (%)**	42.6%		57.4%	
**Chronic cough**	75.6	78.5		
**Chronic sputum**	62	64.2	NS	**182/245**
**Dyspnea MRC stage 0- 1–2 (%)**	73.7	72.7	NS	179/242
**Dyspnea MRC stage 3–4 (%)**	26.3	27.3		
**COPD exacerbation (< 12 months) (%)**	58.2	65.7	NS	**182/245**
**FEV**_**1 **_**(% predicted)**	46.2 ±14.4	52.1 ± 15.9	< 0.001	162/224
**FEV**_**1**_**/FVC post BD**	57.9 ± 13.6	58.9 ± 13.1	NS	170/231
**GOLD stage 2**	42.9%	54.6%		
**3**	39.5%	37.4%	0.026	119/163
**4**	17.6%	8.0%		
**Short- acting ß2 -agonists**	40	53.1	0.010	**170/226**
**BOD index**	2.9 ±1.6	2.6 ±1.6	NS	183/247
**HAD anxiety sub-score (0–21)**	7.1 ± 4.3	9.8 ± 4.3	< 0.001	131/177
**HAD depression sub-score (0–21)**	6.1 ± 4.4	6.6 ± 4.6	NS	128/177
**Q- MAT score (0–20)**	9.3 ±6	8.7 ±5.9	NS	46/93

**Figure 1:**
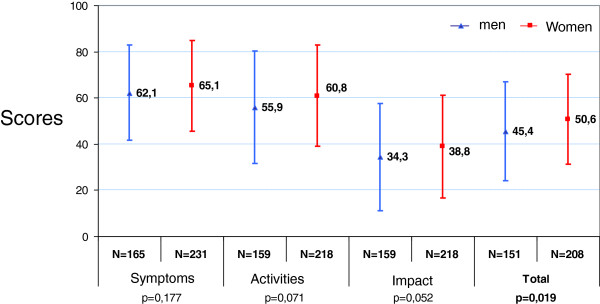
Quality of life: SGRQ total score and subscores.

In active smokers, there was no difference in terms of motivation to quit smoking between men and women (Table 2). Women with COPD had significantly worse quality of life total scores (4 points of difference for clinical relevance) compared with men, and a trend was observed for each domain: symptoms, activities and impacts (Figure 1).

In the multivariate model to determine factors associated with quality of life, we only included variables with less than 25% of data lacking, which decreased the size of the sample.

Regarding factors associated with poor quality of life (Table 3), the following factors were related in the global population: severity of COPD (FEV1), severity of dyspnea, chronic sputum, high number of treatments, previous lower respiratory infections, and higher level of anxiety and depression (HAD). Factors related to poor quality of life were different in women compared with men. Chronic sputum was significantly associated with poor quality of life in women, whereas the number of treatments was associated with it in men.

**Table 3:** Factors determining quality of life (SGRQ-C total score): multivariate analysis

	**Men**	**Women**	**Total**
	**N = 75**	**N = 100**	**N = 175**
**COPD stage (GOLD)**	**4.37** [0.31; 8.43]	**3.07** [−0.89; 7.02]	**3.16** [0.37; 5.94]
	p = 0.035	p = 0.127	p = 0.027
**HAD depression**	**1.84** [1.12; 2.56]	**1.89** [1.36; 2.41]	**1.62** [1.09; 2.15]
**Sub-score**	p < 0.001	p < 0.001	p < 0.001
**MRC dyspnea score**	**17.07** [9.71; 24.44]	**10.73** [4.80; 16.65]	**12.37** [7.56; 17.19]
	p < 0.001	p = 0.001	p < 0.001
**Chronic sputum**	**NS**	**8.33** [3.20; 13.45] p = 0.002	**6.23** [2.22; 10.23] p = 0.003
**Number of treatments**	**3.13** [1.26; 5.00] p = 0.001	**NS**	**1.89** [0.50; 3.28] p = 0.008

**β** (95% IC); analysis done using factors with less than 25% of data lacking.

NS: non significant.

## Discussion

This study was conducted to assess clinical differences by gender in COPD patients and to determine factors related to quality of life in an outpatient setting in France.

Our study describes the clinical profile of COPD in women and adds evidence for gender-related differences adjusted on COPD severity. Women with COPD were younger, had lower BMI, more unemployed and lived more alone than men. Despite the lower severity of the disease as assessed by GOLD staging, women with COPD had a worse quality of life compared to men, and were more impacted by chronic cough.

In the large Muvice Study [14], lower BMI was found in women compared to men, suggesting that COPD may affect nutritional status differently in women. Previously, a high proportion of non-smokers and a lower proportion of active smokers were found in women [14]. However, this was not the case in our study, with a higher proportion of active smokers in women compared to men. This may be due to a change in smoking trends in France, although a selection bias cannot be ruled out.

Skuliem et al. reported a greater proportion of women living alone compared to men [15]. Social support could be included in the management of these patients, in order to improve education about treatment and adherence to it. In turn, this could also help to reduce the anxiety-depression that patients experience [16].

Cardiovascular comorbidities, dyslipidemia, alcoholism and sleep apnea syndrome were more frequent in men. By contrast, osteoporosis, past anxiety and depression were more frequent in women. In the large EPIDEPOC study [17], ischemic heart disease was more frequent in men whereas hypertension, diabetes, anxiety and depression were more frequent in women. Our finding could also reveal an underestimation of cardiovascular disease in women in general practice. A high proportion of anxiety-depression could influence the under-diagnosis of COPD in women, as asthenia could mask dyspnea in women and could be a negative factor of smoking cessation. The impact of osteoporosis could also be important, because of the frequent prescription of oral corticosteroids to treat exacerbation of COPD, which in turn could increase the risk of osteoporosis and complications [18].

There was no difference between men and women regarding the frequency of cough, sputum and dyspnea. However, with regard to the severity of the disease as assessed by FEV1, COPD was less severe in women. In other studies focusing on gender difference in patients with COPD, this was previously observed [8,14,17] and it was also the case in the Uplift study [19].

Finally, despite the lower severity of the disease as assessed by GOLD staging, women with COPD had a worse quality of life as measured by the SGRQ questionnaire compared with men, as reported previously [8,20]. In addition, chronic sputum was significantly associated with poor quality of life in women, whereas it was the number of treatments which was associated with it in men. In a recent meta-analysis by Tsiligianni et al. [7], health status was evaluated by various questionnaires whose pertinence was influenced by several factors. Dyspnea, anxiety and depression were more correlated with worse quality of life than spirometric measurements, suggesting that quality of life should be measured in addition to spirometry in the management of patients with COPD [7].

Our findings suggest that management of COPD should take psychological distress and socioeconomic status into account, particularly in women like those in our population, who were more unemployed than men and lived alone more. It is widely admitted in clinical practice that women feel uneasy about expectorating or eliminating sputum, or simply are unable to do so, but there is no published evidence. Among our COPD patients followed by specialists, there was the same proportion of patients with sputum in women and in men, while this is a key symptom of a lower quality of life mainly in women. Management with physiotherapy could improve quality of life in such women.

Our study had some limitations. This could be due to the design of the study, because the first patient included had to be a woman so as to be sure that as many women as men were included. Secondly, in the final model to determine factors associated with quality of life, we only included variables with less than 25% of data lacking, which decreased the size of the sample. A causal inference cannot be drawn because of the cross sectional design of the study. Our sample was made by patients with COPD referred to pulmonologists, so we cannot generalize our results to the population of COPD patients followed in general practice.

## Conclusions

These findings show that although women experience less airflow limitation, their quality of life in COPD seems more impaired than that of men. Although FEV1 values were higher in women compared to men, COPD had a greater impact on them. It is too restrictive to assess COPD severity only by testing lung function. Assessment of COPD severity could take into account gender, co-morbidities and quality of life in order to obtain a composite severity score. Factors associated with quality of life differ according to gender, with a specific impact of chronic sputum in women. Management of women with COPD should incorporate these specific characteristics, the impact of the disease and gender-specific comorbidities in order to improve their quality of life.

## Abbreviations

COPD: Chronic obstructive pulmonary disease; SGRQ-C: Saint-George Hospital respiratory Questionnaire; HADS: Hospital anxiety and depression scale; QMAT: Motivation to quit smoking; WHO: World Health Organization; BOD index: Body mass, Obstruction, dyspnea index.

## Competing interest

Chantal Raherison received speaker fees and congress invitations from the following companies: Novartis, Astra Zeneca, MundiPharma, Chiesi, Nycomed, Pierre Fabre. Isabelle Tillie-Leblond received speaker fees and congress invitations from the following companies: Novartis, Astra Zeneca, MundiPharma, Chiesi, Nycomed, GlaxoSmithKline. Camille Taillé received speaker fees and congress invitations from the following companies: Novartis, Astra Zeneca, GlaxoSmithKline, MSD, Chiesi, Teva. Anne Prudhomme received speaker fees and congress invitations from the following companies: Novartis, AstraZeneca, MundiPharma, GlaxoSmithKline. Elisabeth Biron received speaker fees and congress invitations from the following companies: AstraZeneca. Cecilia Nocent received speaker fess and congress invitations from the following companies: Astra Zeneca, Novartis, Merck Sharp & Dohme-Chibret, GlaxoSmithKline, ALK. Bénigne Mathieu and Juliette Ostinelli are full time employees of AstraZeneca France, Medical Department.

## Authors’ contributions

All authors were involved in the study design, discussed the results. CR wrote the paper. All authors commented on the revised manuscript. All authors read and approved the final manuscript.

## Pre-publication history

The pre-publication history for this paper can be accessed here:

http://www.biomedcentral.com/1472-6874/14/31/prepub
